# Limitation of Studies on Food Intake and Dietary Pattern in Iran and other Middle East Countries: Lack of Alcohol Intake Assessment

**DOI:** 10.3390/nu9111183

**Published:** 2017-10-28

**Authors:** Shahab Alizadeh

**Affiliations:** Department of Cellular and Molecular Nutrition, School of Nutritional Sciences and Dietetics, Tehran University of Medical Sciences (TUMS), P.O. Box 14155-6446, Tehran P.O. Box 14155-6446, Iran; sh_alizadeh@razi.tums.ac.ir

Alcohol has a major effect on health outcomes of which some are favorable, but the net impact of alcohol on health is detrimental, with an estimated 3.8% of all worldwide deaths and 4.6% of global disability-adjusted life-years attributable to alcohol [1]. Alcohol use is an understudied subject in Iran and other Middle Eastern Countries. A recent study reported that the 12-month prevalence of alcohol use and alcohol-related disorders in Iran is 5.7% and 1%, respectively [2], which is moderate compared with other Middle Eastern countries [3,4]. Nevertheless, the actual prevalence might be higher, as weak surveillance systems and the religious and legal barriers may have resulted in underestimation of the alcohol consumption. This is mostly because of the Muslim majority in this area. Alcohol consumption is firmly banned in Islam. Until recently, the general belief was that alcohol consumption and the related problems are extremely uncommon. This belief, in conjunction with legal and religious prohibitions concerning alcohol, has resulted in neglect and denial of alcohol use and its associated health outcomes [2]. It is notable that recent social changes towards modernization and westernization of lifestyle have led to an increasing prevalence of alcohol consumption in the younger generation of people living in Middle Eastern Countries [5]. Legal sanctions might have acted as obstacles to scientific research on the prevalence of alcohol consumption and its related complications in the community and public health planning to fight against its unfavorable impacts [6]. There are a large number of studies examining the effect of diet on diseases in Middle Eastern countries, but a significant limitation of these studies is a lack of alcohol intake assessment in food questionnaires. However, the food frequency questionnaire (FFQ) which is being used in surveys conducted in Iran has been validated for many foods and nutrients and against multiple diet records or 24-hour recalls in the Iranian population [7,8], but it yields no data concerning alcohol intake.

Considering the prohibition on alcohol which has resulted in the reluctance of individuals to report consumption, reliability could be enhanced by giving official permission for researchers to study alcohol consumption and ensuring participants that there would be no prosecution of their reported alcohol use. By adopting this approach, researchers for the Golestan Cohort Study [9], which was a turning point in investigating alcohol-related diseases in Iran, developed a self-reported questionnaire for alcohol consumption that has provided a reliable and valid measurement in the Iranian population, and is useful for studying the relationship between alcohol use and related diseases in the future.

In conclusion, considering the potential effect of alcohol on health consequences, assessment of alcohol intake in studies exploring the effect of food items and dietary patterns on diet-associated diseases in Iran and other Middle Eastern countries could provide valuable evidence regarding the real social, economic, and health burden of alcohol use on the communities in this region.
